# Diet-induced bacterial immunogens in the gastrointestinal tract of dairy cows: Impacts on immunity and metabolism

**DOI:** 10.1186/1751-0147-53-48

**Published:** 2011-08-09

**Authors:** Guozhong Dong, Shimin Liu, Yongxia Wu, Chunlong Lei, Jun Zhou, Sen Zhang

**Affiliations:** 1 College of Animal Science and Technology, Southwest University, and Key Laboratory of Grass and Herbivores of Chongqing; Beibei, Chongqing, 400716, P. R. China; 2 School of Animal Biology, Faculty of Natural and Agricultural Sciences, University of Western Australia, 35 Stirling Highway, Crawley WA 6009, Australia

**Keywords:** bacterial immunogens, lipopolysaccharide, acute phase proteins, subacute ruminal acidosis, dairy cows

## Abstract

Dairy cows are often fed high grain diets to meet the energy demand for high milk production or simply due to a lack of forages at times. As a result, ruminal acidosis, especially subacute ruminal acidosis (SARA), occurs frequently in practical dairy production. When SARA occurs, bacterial endotoxin (or lipopolysaccharide, LPS) is released in the rumen and the large intestine in a large amount. Many other bacterial immunogens may also be released in the digestive tract following feeding dairy cows diets containing high proportions of grain. LPS can be translocated into the bloodstream across the epithelium of the digestive tract, especially the lower tract, due to possible alterations of permeability and injuries of the epithelial tissue. As a result, the concentration of blood LPS increases. Immune responses are subsequently caused by circulating LPS, and the systemic effects include increases in concentrations of neutrophils and the acute phase proteins such as serum amyloid-A (SAA), haptoglobin (Hp), LPS binding protein (LBP), and C-reactive protein (CRP) in blood. Entry of LPS into blood can also result in metabolic alterations. Blood glucose and nonesterified fatty acid concentrations are enhanced accompanying an increase of blood LPS after increasing the amount of grain in the diet, which adversely affects feed intake of dairy cows. As the proportions of grain in the diet increase, patterns of plasma β-hydoxybutyric acid, cholesterol, and minerals (Ca, Fe, and Zn) are also perturbed. The bacterial immunogens can also lead to reduced supply of nutrients for synthesis of milk components and depressed functions of the epithelial cells in the mammary gland. The immune responses and metabolic alterations caused by circulating bacterial immunogens will exert an effect on milk production. It has been demonstrated that increases in concentrations of ruminal LPS and plasma acute phase proteins (CRP, SAA, and LBP) are associated with declines in milk fat content, milk fat yield, 3.5% fat-corrected milk yield, as well as milk energy efficiency.

## Introduction

Dairy cows are often fed high grain diets to meet the energy demand for high milk production or simply due to a lack of forages at times. As a result, ruminal acidosis, especially subacute ruminal acidosis (SARA), occurs frequently in practical dairy production. It has been recognized that the yield of harmful and toxic substances, such as lactate (particularly the D-isomer), ethanol, histamine, tyramine, tryptamine, and bacterial endotoxin (or lipopolysaccharide, LPS), in the rumen increases as a result of grain-based SARA [1,2]. Other immunogenic virulence factors such as fimbrial adhesins, heat-stable and heat-labile toxins, and inflammatory peptides are also released in the digestive tract due to disturbance in microbial ecology [2]. Among those harmful and toxic substances, the bacterial endotoxin LPS has received a lot of attention because LPS potentially causes systemic immune responses and metabolic changes in the body. However, the other immunogens of bacterial origin induced by feeding high grain diets are attracting attention. This paper reviews the yield and translocation of LPS as well as other bacterial immunogens in the digestive tract and the immune responses and metabolic alterations caused by LPS in dairy cows fed diets containing high portions of grain. The review is based on studies carried out with dairy cows although studies involving beef cattle are also cited where data on dairy cows are lacking.

### Lipopolysaccharide and Other Bacterial Immunogens Released in the Rumen and the Large Intestine

It is widely accepted that free ruminal LPS concentrations increase after grain engorgement, especially during experimentally-induced SARA. In an in vitro fermentation study, Nagaraja et al. [3] found a greater decrease in ruminal pH but a greater increase in free ruminal endotoxin with corn as the substrate than with alfalfa. They also found feeding grain to cows not adapted to grain resulted in higher free ruminal endotoxin, and the endotoxin concentration in the rumen increased by 15 to 18 times within 12 hours after SARA was induced by feeding grain. In the study of Khafipour et al. [4], replacing 21% of the dry matter (DM) of the control diet with a forage to concentrate ratio (F:C) of 50:50 with pellets containing 50% ground wheat and 50% ground barley resulted in grain-based SARA, which exhibited a rise of free rumen LPS concentrations from 28,184 to 107,152 endotoxin units (EU)/mL. Gozho et al. [5] induced SARA in dairy cows by replacing 25% (DM basis) of the total mixed ration containing 44% concentrate with a concentrate made of 50% wheat and 50% barley. In their study, inducing SARA increased free ruminal LPS concentration from 24,547 to 128,825 EU/mL. A study by Emmanuel et al. [6] showed ruminal LPS content increased in dairy cows receiving 30% or 40% barley grain (5,021 and 8,870 ng/mL, respectively) compared with those fed no grain or 15% barley grain (654 and 790 ng/mL, respectively). When dairy cows were fed a control diet containing 70% of forage and 30% mixed concentrates (DM basis), a high grain diet (38% wheat-barley pellets, 32% mixed concentrates, and 30% of forages), or a diet containing alfalfa pellets (45% of mixed concentrates, 32% of alfalfa pellets, and 23% of other forages), the ruminal LPS concentrations were 8,333, 124,566, and 18,425 EU/mL, respectively [7]. Andersen et al. [8] reported free ruminal LPS concentration in non-lactating cows fed with hay was only 118 to 148 EU/mL, whereas increasing concentrate feeding resulted in a free ruminal LPS concentration of 1,600 EU/mL. According to Gozho et al. [9], when beef cattle were fed diets with different F:C (100:0, 79:21, 59:41, 39:61, and 24:76), free ruminal LPS concentrations increased curvilinearly when the proportions of concentrate in the diet increased. They also found the following relationship between dietary concentrate proportion (x, %) and ruminal LPS concentration (y, log_10 _EU/mL): y = 0.00009x^2^+ 0.0023x + 3.8071 (R^2^= 0.99).

LPS is the component of cell wall of Gram-negative bacteria that are predominant bacterial group in the rumen. A decline in ruminal pH during SARA causes death and cell lysis of Gram-negative bacteria, resulting in an increase in free ruminal LPS concentration [1-3]. However, rapid growth of Gram-negative bacteria can also result in the shedding of LPS in the rumen [1,10]. LPS released during growth of bacteria may account for as much as 60% of that released in the rumen [11]. During rapid growth, autolytic enzymes are required to help cells expand and grow. However, excessive autolytic activity can lead to bacterial cell apopotosis and lysis. It was reported that the autolysis of *Fibrobacter succinogen *during rapid growth was 10 times higher than that during the stationary phase [12]. It is possible that a certain range of ruminal pH after grain engorgement is conducive to bacteria rapid growth, which leads to an increase in free ruminal LPS concentration. It was shown that high-grain diets which were normally associated with SARA resulted in much higher numbers of *E. coli*, a Gram-negative bacterium, in the rumen [13]. The results of a study by Khafipour et al. [14] showed that the abundance of *E. coli *in the rumen was highly correlated with the severity of SARA and the degree of inflammation, and *E. coli *were a major contributor to the rumen LPS pool. According to Nagaraja and Titgemeyer [1], provision of additional grain in the diet could trigger rapid growth of starch/sugar fermenting Gram-negative bacteria, such as *Prevotella spp*., *Ruminobacter amylophilus*, *Succinimonas amylolytica*, and *Succinivibrio dextrinosolvens*. It is suggested that the increased shedding of LPS during the early hours postfeeding is due to rapid growth of Gram-negative bacteria, and the later release of LPS is because of bacterial cell lysis as a result of the lower pH in the rumen.

LPS may also be produced in other parts of the gastrointestinal tract. During grain-based SARA, more starch may enter the lower digestive tract including the ileum and the large intestine where the metabolite profile may resemble that in the rumen and LPS may be produced in a significant amount. In a recent study by Li et al. [7], a grain-pellet induced SARA challenge in dairy cows significantly increased cecum LPS content (128,410 EU/mL) compared with control (18,289 EU/mL). Interestingly, in their study an alfalfa-pellet induced SARA challenge did not significantly increase cecum LPS content (15,631 EU/mL) compared with control (18,289 EU/mL) although the alfalfa-pellet induced SARA challenge did significantly reduce cecum pH. They pointed out feeding forage pellets did not increase the content of starch in the diet. Therefore, the increase in cecum LPS could be attributed to more starch entering the lower gut rather than the acidic disturbance in the lower gut.

The starch entering the lower gut may be conducive to the growth of Gram-negative bacteria, which may result in an increase in LPS as described above. In an interesting study by Diez-Gonzalez et al. [13], the total *E. coli *count was only 2 × 10^4 ^cells per gram of colonic digesta in cattle fed either hay or fresh grass (pasture), whereas the total *E.coli *population was 6.3 × 10^6 ^viable cells per gram of colonic digesta in cattle fed moderate amounts of grain (60% of DM). When cattle were fed more than 80% grain, the *E. coli *count was further increased. Grain feeding (90% grain in the diet) increased the numbers of anaerobic bacteria in the colon by 1000-fold.

It is also worth mentioning that other immunogenic virulence factors may be produced by pathogenic *E. coli *after cattle are fed more grain. Although not all *E. coli *are pathogenic when the amount of grain in the diet is increased, there is a great possibility that pathogenic strains are harbored by cattle [15,16]. For example, ruminants, especially cattle, are the major reservoir of Shiga toxin-producing *E. coli *(STEC), and more than 435 serotypes of STEC have been recovered from cattle [16]. Even healthy cattle can shed a significant amount of STEC which has led to frequent infections in humans around the world [16].

### Translocation of the Bacterial Immunogens in the Digestive Tract

Endotoxin produced in the digestive tract can be translocated into the bloodstream, thus the concentration of blood LPS increases [4,17]. When Khafipour et al. [4] replaced 21% of the DM of the control diet (F:C = 50:50) with pellets containing 50% ground wheat and 50% ground barley, the concentrations of both ruminal and blood LPS increased. It was also observed in other studies that SARA led to a rise of blood LPS concentrations [18,19]. Although there is a general agreement that LPS translocation into blood occurs as a result of grain-induced SARA, the translocation sites remain unknown. There is no direct evidence that free ruminal LPS during grain-induced SARA is translocated across the rumen wall into circulating blood. Rumen epithelium has a multilayer structure whose tight junctions are located in the middle layers, stratum grannulosum, and spinosum [20]. Although the external layer of rumen epithelium has no tight junctions, it may have up to 15 cell layers, which can limit the permeability of LPS, a large molecule [21]. In the in vitro study of Emmanuel et al. [22], LPS increased the permeability of the rumen wall, and the rate of LPS translocation across the rumen wall was numerically higher at pH 5.5 than at other pH levels. However, the concentration of LPS added to the mucosal side tissues in their study was 500 μg/mL that was 50 times more than that of free rumen LPS during grain-induce SARA [4,6], which might have disrupted the rumen epithelial structure and impaired the barrier function of rumen epithelium to a greater extent than what would have occurred at the physiological state. Studies were conducted to investigate ruminal absorption of endotoxin in steers by administering ^51^Cr-labeled *E. coli *endotoxin into the rumen [23,24]. The results showed no absorption either through lymph (the thoracic duct) or blood (the portal vein) occurred in any of the steers, whether forage-fed (100% alfalfa hay diet), grain-fed (92% concentrate diet based on sorghum grain), or ruminally acidotic. A recent study by Khafipour et al. [14] showed that LPS in the rumen was not highly correlated with the severity of SARA and the degree of inflammation. Therefore, it seems the ruminal epithelium is impermeable to endotoxin at the physiological state unless the rumen epithelial structure is disrupted to a greater extent. Thus it is likely that LPS translocation occurs mainly in the intestines. Epithelium in the intestines is of a monolayer structure with tight junctions at the apical pole of the cells. In the study of Chin et al. [25] using intestinal epithelial cell lines, an abnormal increase in luminal LPS induced cell apoptosis, disrupted tight junction protein zonula occludens-1, and enhanced epithelial permeability in a dose and time dependent manner by increasing the production of nitric oxide. The results of a study by Cetin et al. [26] demonstrated that the pH regulatory system of enterocytes was impaired by LPS through inhibition of sodium-proton pumps under extracellular acidosis conditions, which resulted in cytoplasmic acidification and cellular dysfunction.

LPS flowing to the intestines could be detoxified in the duodenum by bile acid [27]. However, since the rumen is an immense LPS source, LPS entering the intestines may not be completely detoxified in the duodenum and may be translocated into circulation across the intestines. In addition, a source of LPS which is translocated into blood circulation may be produced originally in the lower gut. In this case, the LPS production would not be pH dependent, but starch dependent. The bypass starch which reaches the ileum and large intestine may result in a change of microbiota there and thus the release of LPS in a manner described previously for the rumen. In fact, many forms of starch can pass through the pregastric stomach (rumen) to the intestines [28]. Allen [29] indicated that up to 44% of starch in the diet can be digested postruminally. A recent study by Li et al. [7] demonstrated a grain-pellet induced SARA challenge in dairy cows significantly increased LPS production in the cecum compared with control. However, in their study an alfalfa-pellet induced SARA challenge did not increase cecum LPS release compared with control because feeding forage pellets did not increase the content of starch in the diet. In another study by Khafipour et al. [30], they replaced chopped alfalfa hay with alfalfa pellets to induce low pH in the rumen of dairy cows without any changes in the starch content or the F:C ratio of the diets. Although free rumen LPS concentration increased by 3.5-fold, this increase was not accompanied by increases in LPS and the acute phase proteins such as LPS binding protein (LPB), serum amyloid-A (SAA), and haptoglobin (Hp) in peripheral circulation. They suggested that LPS translocation across rumen epithelium did not occur, neither did the translocation across intestinal epithelium. The reason could be that either LPS produced in the rumen during SARA failed to penetrate the rumen wall, or feeding alfalfa pellets did not increase additional starch flow to the intestines to modify the profile of bacterial communities of the lower gut. Collectively, LPS can be produced in the lower gut following feeding a high grain diet, thus it may disrupt the barrier function of monolayer epithelial structure of the intestines as described above. Although an in vitro study with an Ussing chamber system demonstrates that LPS goes through the colon of ruminant animals [22], in vivo studies are warranted to verify that the barrier failure and subsequent LPS translocation occur in the lower gut after a grain-based challenge.

### The Immune Response to the Bacterial Immunogens

After LPS is translocated into the blood stream, immune responses are subsequently caused by circulating LPS [31], and the systemic effects include an increase in the blood concentrations of neutrophils [3] and the acute phase proteins, such as SAA, Hp, and LPB [4]. The increase of acute phase proteins in the systemic circulation is a non-specific acute phase response activated by endotoxin. The translocation of LPS into the systemic circulation stimulates the release of proinflammatory cytokines such as interleukin-1 (IL-1), IL-6, and tumor necrosis factor-α (TNF-α) by mononuclear phagocytes, and these mediators in turn result in enhanced secretion of acute phase proteins from hepatocytes [32].

When the proportion of concentrate in steer diets was enhanced from 0% to 76%, ruminal LPS concentrations were continually increasing, and the acute phase proteins (SAA and Hp) in plasma also kept rising [9]. Many studies have confirmed that increases in ruminal LPS content result in a rise of the acute phase proteins such as SAA [5,6,33], LBP [6,21], and C-reactive protein (CRP) [6,33] in blood of dairy cows. Therefore, the circulating LPS resulting from feeding increasing proportions of grain to dairy cows can lead to proinflammatory reactions, and in turn milk production in dairy cows can be adversely affected. It was demonstrated that increases in concentrations of ruminal LPS and plasma acute phase proteins (CRP, SAA, and LBP) were associated with declines in milk fat content, milk fat yield, 3.5% fat-corrected milk yield, as well as milk energy efficiency [33]. The results of a study by Khafipour et al. [4] also showed deceased milk yield, milk fat content, and milk fat yield in dairy cows in response to the increase of grain amount in the diet that triggered an inflammatory response. According to the study by Zebeli and Ametaj [33], milk fat content, milk fat yield, and 3.5% fat-corrected milk yield are negatively correlated with the concentration of plasma CRP in dairy cows fed graded barley grain (0%, 15%, 30%, 45%).

Although studies on diet-induced bacterial immunogens are focused on LPS, it does not mean the inflammation in relation to grain-induced SARA is caused solely by LPS. Other immunogenic virulence factors in the digestive tract following feeding a high grain diet may have contributed to the inflammation which has been observed in many studies on grain-induced SARA in dairy cattle. For example, a variety of virulence factors that have the potential to cause inflammation are produced by *Escherichia coli spp*., as well as other members of the *Enterobacteriacae *[34]. These virulence factors include fimbrial adhesins, heat-stable and heat-labile toxins, and inflammatory peptides. Gyles [16] has reviewed *E. coli *virulence factors in relation to a number of genes and gene products produced by *E. coli *that can elicit inflammation in dairy cattle. High grain feeding can promote rapid growth of *E. coli *including the pathogenic *E. coli *in the digestive tract of dairy cattle as described previously, which could result in release of many of the immunogenic virulence factors. For example, low ruminal and intestinal pH due to high grain feeding increases the risk of shedding enterohemorrhagic *E. coli *such as 0157:H7 which can produce a number of immunogenic virulence factors [35].

### Impact of the Bacterial Immunogens on Metabolism

Translocation of endotoxin into the bloodstream can also lead to metabolic alterations and perturb blood metabolites by inducing a systemic inflammatory response [36]. Blood glucose was enhanced accompanying an increase of blood LPS during a grain-based SARA challenge [4]. Ametaj et al. [36] reported that both glucose and nonesterified fatty acid (NEFA) concentrations in blood were increased after including high proportions of barley grain into the diet of dairy cows. Increases in blood glucose and NEFA may adversely affect feed intake of dairy cows. In fact, many studies showed feed intake decreased following occurrence of SARA [37-39], and reduced feed intake is a consistent sign of SARA in both dairy cows [2,40,41] and beef cattle [42,43]. When dairy cows were fed diets containing different proportions (0, 15, 30 and 45%, DM basis) of barley grain, feed intake was 32.6, 32.9, 27.34 and 25.18 kg/d, respectively [6]. It can be seen that raising barley grain proportion from 0 to 15% did not affect feed intake, whereas feed intake was reduced significantly after barley proportions reached 30 and 45%. Interestingly, the corresponding DM intake in their study was 13.33, 15.28, 14.68 and 16.04 kg/d, respectively, and increasing the amount of barley grain in the diet significantly increased DM intake. In the study of Emmanuel et al. [6], barley grain contained higher DM, thus DM intake was increased when barley grain was included into the diet of dairy cows. In contrast, in the study of Khafipour et al. [4], replacing 21% of the DM of the control diet (F:C = 50:50) with pellets containing 50% ground wheat and 50% ground barley depressed DM intake by 15% compared with the control group. The barley proportions and DM contents in the diets of the aforementioned two studies were different, which might serve as an explanation for the differences in DM intake between the two studies.

The lower feed intake cannot be simply attributed to increases in blood glucose and NEFA after increasing grain amount in the diet. Feeding dairy cows high-grain diets rich in rapidly fermentable carbohydrates will lead to increased yield of volatile fatty acids, especially propionate in the rumen, and its absorption into the bloodstream [44]. The absorption of propionate into blood circulation or its effects on rumen receptors may result in decreased feed intake in cows fed high grain diets [6]. In addition, deceased feed intake with increasing the amount of grain in the diet may be due to enhanced release of endotoxin and other bacterial immunogens in the digestive tract and their translocation into blood. Increased endotoxin concentrations in the bloodstream will lead to release of cytokines such as IL-1, IL-6, and TNF-α due to activation of macrophages [32], and IL-1 and TNF-α can suppress feed intake in different species [45].

After increasing the proportions of grain in the diet, diurnal patterns of plasma β-hydoxybutyric acid, cholesterol, and minerals (Ca, Fe, Zn, and Cu) were perturbed [36,46]. When dairy cows were fed diets containing barley grain at 0, 15, 30 and 45% (DM basis), plasma β-hydoxybutyric acid and cholesterol concentrations decreased with increasing barley proportions in the diet [36]. Increasing the amount of barley grain in the diet was also associated with quadratic responses of plasma Ca, Fe and Zn concentrations [46]. Cows fed the greatest amount of barley grain (i.e., 45%) had the lowest concentrations of Ca, Fe and Zn in the plasma, whereas the highest concentrations of Ca, Fe and Zn were observed in the plasma of cows fed the 15% grain-based diet. Plasma Cu concentrations were not affected by the amount of barley grain in the diet. Their study revealed that the increase in rumen endotoxin in response to high grain diet, and the resulting increases in plasma SAA and CRP, were strongly correlated with fluctuations of plasma minerals [46]. The changes in plasma concentrations of metabolites and minerals as a result of increasing grain amount in the diet may have an effect on the health and productivity of dairy cows. A detailed discussion on this issue is beyond the scope of this paper, and a review paper published recently by Ametaj et al. is available [47].

As discussed previously, endotoxin and other bacterial immunogens which are translocated into blood will elicit systemic inflammatory responses. Under the circumstances, nutrients will be directed to support proinflammatory events. The redirection or repartition of nutrient use in addition to a low nutrient supply due to depressed low feed intake will decrease nutrient flow to the mammary gland. Furthermore, when endotoxin and other immunogens are transported to the mammary gland through blood circulation, metabolism in this tissue can be affected. On the one hand, the bacterial immunogens that enter the mammary tissue will elicit a local immune response, and more nutrients or precursors of milk components will be directed to support immune response processes including the synthesis of immune molecules. The repartition of precursor use will lead to less precursors being used for synthesizing milk components, resulting in reduced synthesis of milk components. On the other hand, the bacterial immunogens entering the mammary tissue may directly exert harmful effects on the mammary epithelial cells, which may lead to depressed functions and proliferation of the epithelial cells and increased cell apoptosis. Pieces of evidence pinpoint the suppressive effects of LPS on key enzymes, such as fatty acid synthetase and acetyl-CoA carboxylase which are related to de novo fatty acid synthesis [48,49] in the mammary tissue and down-regulation of the activity of lipoprotein lipase [50] which is involved in the uptake of fatty acids for incorporation into milk fat [51]. Moreover, LPS in the mammary tissue will activate neutrophils and activated neutrophils are able to produce a large quantity of bactericidal molecules such as reactive oxygen species that have been associated with tissue damage. It was demonstrated in vitro that activated blood neutrophils had a cytotoxic effect on bovine mammary epithelial cells [52] potentially through the release of reactive oxygen species such as hydroxyl radicals [53].

## Conclusions

Feeding dairy cows diets containing high proportions of grain can lead to release of bacterial immunogens such as LPS in a large amount in the digestive tract. LPS can be translocated into blood due to possible alterations of permeability and injuries of the epithelial tissue of the digestive tract (particularly the lower gut). As a result, immune responses are caused by circulating LPS, which include increases in the concentrations of neutrophils and the acute phase proteins in the bloodstream. Changes in blood concentrations of metabolites and minerals were also observed, which indicates metabolic alterations occur following the entry of endotoxin into blood. The bacterial immunogens can also lead to reduced supply of nutrients for synthesis of milk components and depressed functions of the epithelial cells in the mammary gland. The immune responses and metabolic alterations caused by circulating bacterial immunogens will exert an effect on milk production. Results have shown that increases in concentrations of ruminal LPS and plasma acute phase proteins (CRP, SAA, and LBP) are associated with declines in milk fat content, milk fat yield, 3.5% fat-corrected milk yield, as well as milk energy efficiency.

## Competing interests

The authors declare that they have no competing interests.

## Authors' contributions

GD and SL conceived the overall idea of the review article and wrote the manuscript. YW, CL, JZ and SZ provided ideas and participated in discussions for writing the review. All authors read and approved the manuscript.
